# Wheat ergot fungus-derived and modified drug for inhibition of intracranial aneurysm rupture due to dysfunction of TLR-4 receptor in Alzheimer’s disease

**DOI:** 10.1371/journal.pone.0279616

**Published:** 2023-01-19

**Authors:** Sandip Debnath, Devesh Sharma, Somdatta Yashwant Chaudhari, Ritika Sharma, Amir Afzal Shaikh, Rahul Subhash Buchade, Kavindra Kumar Kesari, Abdel-Fattah M. Abdel-Fattah, Mohammad Algahtani, Mayyadah Mheidat, Rawidh Alsaidalani, Tapas Paul, Amany A. Sayed, Mohamed M. Abdel-Daim

**Affiliations:** 1 Department of Genetics and Plant Breeding, Institute of Agriculture, Visva-Bharati University, Sriniketan, West Bengal, India; 2 Department of Biochemistry, ICMR - National JALMA Institute for Leprosy & Other Mycobacterial Diseases, Agra, Uttar Pradesh, India; 3 Department of Pharmaceutical Chemistry, Progressive Education Society’s Modern College of Pharmacy, Nigdi, Pune, India; 4 Department University Institute of Pharma Sciences, Chandigarh University, Chandigarh, India; 5 Department of Pharmaceutics, SCES’s Indira College of Pharmacy "Niramay", Tathwade, Pune, Maharashtra, India; 6 Department of Applied Physics, School of Science, Aalto University, Espoo, Finland; 7 Pharmacology Department, Faculty of Veterinary Medicine, Suez Canal University, Ismailia, Egypt; 8 Department of Laboratory & Blood Bank, Security Forces Hospital, Meca, Saudi Arabia; 9 Medicine Program, Batterjee Medical College, Jeddah, Saudi Arabia; 10 Department of Pharmaceutical Sciences, Pharmacy Program, Batterjee Medical College, Jeddah, Saudi Arabia; 11 Zoology Department, Faculty of Science, Cairo University, Giza, Egypt; Gauhati University, INDIA

## Abstract

**Background:**

Alzheimer’s disease (AD) is a form of dementia that strikes elderly people more frequently than it does younger people. The cognitive skills and memory of Alzheimer’s sufferers continue to deteriorate over time. Recent studies have shown that patients with AD have greater amounts of inflammatory markers in their bodies, which suggests that inflammation occurs early on in the progression of the disease. There is a possibility that Aß oligomers and fibrils can be recognised by TLRs, in addition to the microglial receptors CD14, CD36, and CD47. When Aß binds to either CD36 or TLR4, it sets off a chain reaction of inflammatory chemokines and cytokines that ultimately results in neurodegeneration. Diabetes and Alzheimer’s disease have both been recently related to TLR4. The activation of TLR4 has been connected to a variety of clinical difficulties that are associated with diabetes, in addition to the internal environment of the body and the microenvironment of the brain. TLR4 inhibitors have been shown in clinical investigations to not only lessen the likelihood of getting sick but also to increase the average longevity.

**Result:**

In this work we used molecular docking and molecular dynamics modelling to investigate the effectiveness of FDA-approved antidiabetic plant derived drugs in combating the TLR4 receptor. Molecular docking experiments were used to make a prediction regarding the most important interactions involving 2-Bromoergocryptine Mesylate. With a binding affinity of -8.26 kcal/mol, it stood out from the other candidates as the one with the greatest potential. To verify the interaction pattern that takes place between 2-Bromoergocryptine Mesylate and the TLR4 receptor, a molecular dynamic simulation was run at a time scale of 150 nanoseconds. Because of this, 2-Bromoergocryptine Mesylate was able to make substantial contact with the active site, which led to increased structural stability during the process of the complex’s dynamic development.

**Conclusion:**

As a result of this, the results of our research may be relevant for future research into the efficacy of 2-bromoergocryptine mesylate as a potential lead treatment for TLR4 receptors in intracranial aneurysm rupture in AD.

## Introduction

AD sufferers gradually lose their capacity to carry out even the most basic of tasks as the disease progresses. To put it simply, AD is the most common form of dementia in those aged 76 and up [1]. Several potential risk factors for developing AD have recently been identified. These include amyloid beta (Aß) and tau-related pathological alterations. The Aß cascade hypothesis states that glial cells, of which microglia and astrocytes are examples, activate the immune system in response to Aß deposition. New research suggests inflammation has a role in the onset of illness in people with mild cognitive impairment [2, 3]. As a further point, tissue microarrays from people with neurological disorders like AD showed an increase in the quantities of inflammatory components. This means that inflammatory markers can be used to demonstrate a link between AD and its onset at an earlier age [4]. Microglial receptors such as CD14, CD36, CD47, and Toll-like receptors may bind Aß oligomers and fibrils. When a beta molecule attaches to CD36 or TLR4, it triggers the production of inflammatory chemokines and cytokines. More neuronal death happens in AD hotspots as a result of this inflammation has been linked to hyperglycemia, insulin resistance, oxidative stress, and inflammation [5, 6]. It appears that this connection holds true for all of these diseases [6, 7]. Each of these factors has the potential to directly influence Aß and tau problems and to set off a cascade of inflammatory stress responses that may ultimately result in neurodegeneration, as is seen in AD. All of these factors may also contribute to neurodegeneration by setting off a chain reaction of inflammatory stress responses. Hyperglycemia, also known as high blood sugar levels, hyper insulinemia, and insulin resistance are all indicators of type 2 diabetes [7, 8]. Type 2 diabetes is the most common form of the disease, affecting the vast majority of people. It’s a chronic metabolic disorder that’s on the rise all across the globe [9]. Evidence suggests that the TLR4 pathway plays a critical role in the association between diabetes and AD. Insulin resistance has been linked to chronic TLR4 activation [10]. Many complications of diabetes have been connected to TLR4-mediated chronic inflammation [11]. In addition to altering the microenvironment of the brain, TLR4-mediated chronic inflammation affects the rest of the body. Long-term TLR4 activation leads to Aß accumulation in the brain, which is a characteristic of the illness, but it may be beneficial in the early stages of AD by scavenging Aß. In the earliest stages of AD, activation of TLR4 may aid in scavenging Aß [12–14]. Multiple studies employing well-known TLR4 inhibitors have connected TLR4 activation to AD and type 2 diabetes. Reducing the overactive inflammatory response has been shown in these studies to have beneficial effects on health and lifespan. These results suggest that TLR4 inhibitors may have therapeutic potential [13, 14]. The repurposing anti-diabetic drugs as a long-term remedy to the condition with which they are associated is evidently an accurate choice. In this work, we established the capacity of presently available diabetic drugs to bind to TLR4 and block it from activating using computational biology. This was finished in harmony with the standards of the US Food and Drug Administration [12, 13, 15]. Therefore, a exceptional technique of treating AD with a single drug has been revealed. 2-Bromoergocryptine Mesylate is a semisynthetic alkaloid derivative from ergotamine alkaloid from fungus *Clevicepspurpurea* which is then modified and used as 2-Bromoergocryptine Mesylate here in this work this drug have shown a good efficacy and compactness against the target protein.

## Methodology

### Target preparation

TLR4 (**Pdb ID: 4G8A**) crystallographic structure was identified in the Protein Data Bank’s structural database (https://www.rcsb.org/structure/4G8A), and the structure was imported into a molecular editor with an open-source license (Discovery studio visualizer 4.0). The assembly was hoarded in PDB format after co-crystal ligands and heteroatoms were aloof. The UCSF Chimera employed the sharpest descent to select 1000 steps, then used the conjugate gradient of energy minimization tactic to optimize the structure. sdf2-Bromoergocryptine Mesylate was obtained through PubChem (https://pubchem.ncbi.nlm.nih.gov/) (**PubChem ID: 5284352**). The DS visualizer was used to import this dataset, which was then exported as .PDB files.

### Virtual screening of compounds

The compounds were based on previously discovered antidiabetic medications that we used in our inquiry against the TLR4 receptor. Initially, AutoDockVina 4.2.6 was used to test the compounds against our target protein, 4G8A. The anti-diabetic medication known as 2-bromoergocryptine mesylate (**PubChem ID: 5284352**) gets the best dock score out of all of these different options.

### Molecular docking analysis

Using AutoDockvina for macromolecular screening with TLR4 (**Pdb ID: 4G8A**), we discovered that 2-Bromoergocryptine Mesylate (**PubChem ID: 5284352**) has a high dock score. The receptor protein was constructed so that docking may take place using the AutoDock MGL tool version 1.5.6. The receptor grid was built from residues near 2.22, where 2-Bromoergocryptine Mesylate is bound to the co-crystal. The MGL programme accessed the PDBQT files containing the proteins. The AMBER ff4 force field was included into the TLR4 structures, and docking investigations with the ligands of interest were performed, all after the required crystallisation temperature had been adjusted using the steepest descent approach (1000 steps). Protonation states were checked for neutralisation before engaging in an interaction experiment. Autodock, in its version 4.2.6, was used for the molecular docking analyses. Polar hydrogen bonds were coupled with other hydrogen bonds using Kollman and Gastieger charges. The receptor and ligand molecules were saved in pdbqt format after the nonpolar hydrogens were combined. With X = 30, Y = 46, and Z = 36 with a spacing of 2.2, an equilateral grid box was generated. Docking experiments were performed using the Lamarckian Genetic Algorithm (LGA) to determine the binding free energy that resulted in the lowest binding energy (G).

### Molecular dynamics simulations

Using the software programme Desmond, which was developed by Schrodinger LLC, a simulation of molecular dynamics was run for a duration of 150 ns. The simulation was carried out using Desmond [16]. Research on docking was the very first thing that needed to be done in order to get started on the process of building molecular dynamics simulations incorporating protein and ligand complexes. This was necessary in order to begin the process. When carried out in static settings, molecular docking studies have the potential to precisely anticipate the state of ligand binding. The integration of the classical equation of motion is a common method used in MD simulations for tracking the motions of atoms over the course of time. This method is referred to as the integration of the classical equation of motion. The significance of this might be attributed to the fact that docking provides a fixed representation of a molecule’s binding posture in the active site of a protein [15, 17–20]. Simulations were utilised so that an investigation could be conducted into the physiological condition of the ligand binding process. Maestro or the Protein Preparation Wizard were utilised since it was found that the protein–ligand combination needed to be fine-tuned and shrunk. The residues numbered 16 and 17 were involved in this determination. The systems were built with the assistance of a piece of software called System Builder. The orthorhombic box model of the solvent was utilised in all three places so that the transferrable intermolecular interaction potential could be calculated (TIP3P). A force field that was created from OPLS 2005 was utilised in the simulation that was carried out. The models were rendered inactive by utilising counter ions in the process. The physiological circumstances of the experiment were imitated with the help of NaCl, which was administered at a concentration of 0.15 M. The simulation was run with the temperature set at 300 kelvin and the pressure set at one atmosphere throughout the entire process. A larger degree of adaptability was made available to the models in the moments leading up to the start of the simulation. The reliability of the simulation was determined by examining the degree to which the root mean square deviation (RMSD) of the protein and the ligand varied during the duration of the simulation. At 1500-ps intervals, trajectories were saved so that they could be studied afterwards.

## Results and interpretations

### Molecular docking

**Fig 1:**
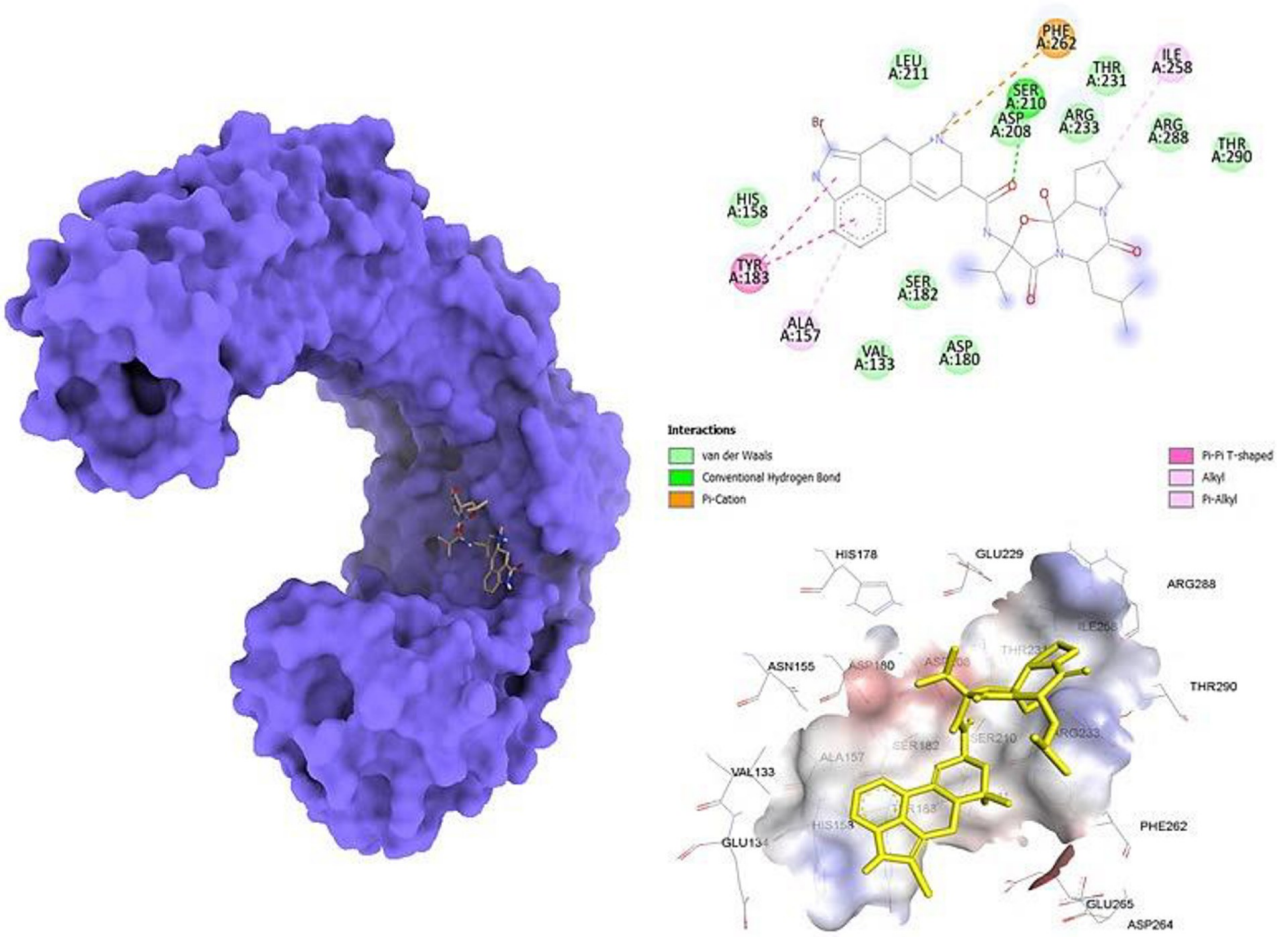
The TLR4-2-Bromoergocryptine Mesylatemesylate complex 3D structure, as well as the 3D and 2D interaction diagram. The left panel shows a 2D interaction plot of 2-Bromoergocryptine Mesylate and TLR4. The Pi-Alkyl bond is shown by pink dashed lines, whereas residues wrapped inside a light green sphere are thought to be involved in Van der Waals interactions. The binding cavity of the 2-Bromoergocryptine Mesylate molecule is shown in the center panel of TLR4, while the right panel shows a zoomed-out binding pocket with amino acid residues surrounding the molecule.

We are seeking to repurpose current diabetes drugs to target the TLR4 enzyme, which has been associated to Parkinson’s disease. The phrase "molecular docking" refers to the act of bringing two molecules closer together. A computer simulation of 17 different medications was used to estimate the TLR4-binding affinity of each of the drugs (as depicted in Table 1). In order to dock a protein-ligand complex, the Genetic Algorithm (GA) was used using the following parameters: 1500 generations, 20000 populations, 3000000 maximum evaluations, and a maximum evaluation count of 27000. During the subsequent docking studies, the Lamarckian Genetic Algorithm (LGA) was used with the purpose of locating the protein-ligand complex that had the lowest binding free energy (G). In conclusion, the inhibitory concentration of the TLR4-2-Bromoergocryptine Mesylatecomplex is calculated to be 782.55 m, the free energy of binding is calculated to be -9.6 kcal/mol, and the total internal energy is calculated to be 0.75 kJ/mol. Additionally, the TLR4-2-Bromoergocryptine Mesylatecomplex has a torsion energy that is calculated to be 0.03 kJ/mol. 2-Bromoergocryptine Mesylatewas shown to be the most effective antidiabetic ligand among all of those tested. The structure of 2-Bromoergocryptine Mesylate, as well as its complex with TLR4, and its 2D and 3D interaction diagrams, are revealed in Fig 1.

**Table 1:** 10 ligands with the most auspicious binding affinity with TLR4 was calculated by molecular docking analysis.

Ligand	Binding Affinity(kcal/mol)
Alogliptin benzoate	-6.8
**2-Bromoergocryptine Mesylate**	***-8*.*26***
Canagliflozin	-7.3
Chlorpropamide	-6.6
Empagliflozin	-7.7
Glyburide	-7.9
Linagliptin	-5.9
Miglitol	-5.8
Nateglinide	-6.9
Pioglitazone	-7.5
Metformin hydrochloride	-4.9

### Molecular dynamic simulations

Calculating the usual change in atom location with respect to a reference frame requires the use of the root mean square deviation (RMSD) statistic. During the process of computing, each individual frame of the trajectory was taken into account. The following is the RMSD for frame x:t_ref_ is the reference time (usually, the initial frame is used as the reference and is regarded to be time t = 0); N is the number of atoms in the atom selection; and ‘r’ is the position of the selected atoms after superimposing on the reference frame in frame x at time tx. Each frame of the simulation trajectory is subjected to the same technique [21]. The Root Mean Square Fluctuation (RMSF) is an excellent method for detecting local changes in the protein chain. The RMSF of Residue I is as follows:

$$RMSF_{i}=\sqrt{\frac{1}{T}\sum_{t=1}^{T}<(r_{i}^{\prime }(t))-r_{i}(t_{ref}){)}^{2}>}$$

As shown by the angle brackets, it is widely assumed that the square distance indicates an average of all of the atoms in the residue. The letter T represents the amount of time spent calculating the RMSF, tref represents the reference time, and ri represents the position of the residue. The symbol r’ will precisely represent the locations of the atoms in the residue I after executing a superposition on the reference. The simulations of Desmond’s voyages were thoroughly examined. The RMSD and RMSF, as well as the protein–ligand interactions, were calculated using MD trajectory analysis. Protein standard deviation is as follows: The graphs show how the relative standard deviation (RMSD) of a protein has changed over time (left Y-axis). After all of the protein frames have been aligned on the backbone of the reference frame, the root mean square deviation (RMSD) is calculated.

During the simulation, it is critical to study the protein’s relative mean square deviation (RMSD), since it may reveal information on the protein’s molecular structure. If the simulation has reached equilibrium, the RMSD analysis can determine whether or not the final fluctuations are centered around an average thermal structure. Changes on the order of 1–3 are thought to be acceptable for proteins that are very small and spherical in form. Larger changes, on the other hand, indicate that the protein is altering form while the simulation is running. The system has not yet achieved equilibrium, and the simulation most likely did not persist long enough to finish the inquiry. The relative mean square deviation of the ligand in reference to the protein and the binding pocket, which can be found on the graph’s right axis, may be used to evaluate its stability in relation to the protein and the binding pocket. After the protein-ligand complex has been aligned on the reference protein backbone, the relative mean square deviation of the ligand is presented. The RMSD of the ligand heavy atoms is then calculated, and the ligand RMSD is shown to the user. When the reported values exceed the protein’s RMSD, the ligand has most likely migrated away from the location where it was initially bound.

**Fig 2:**
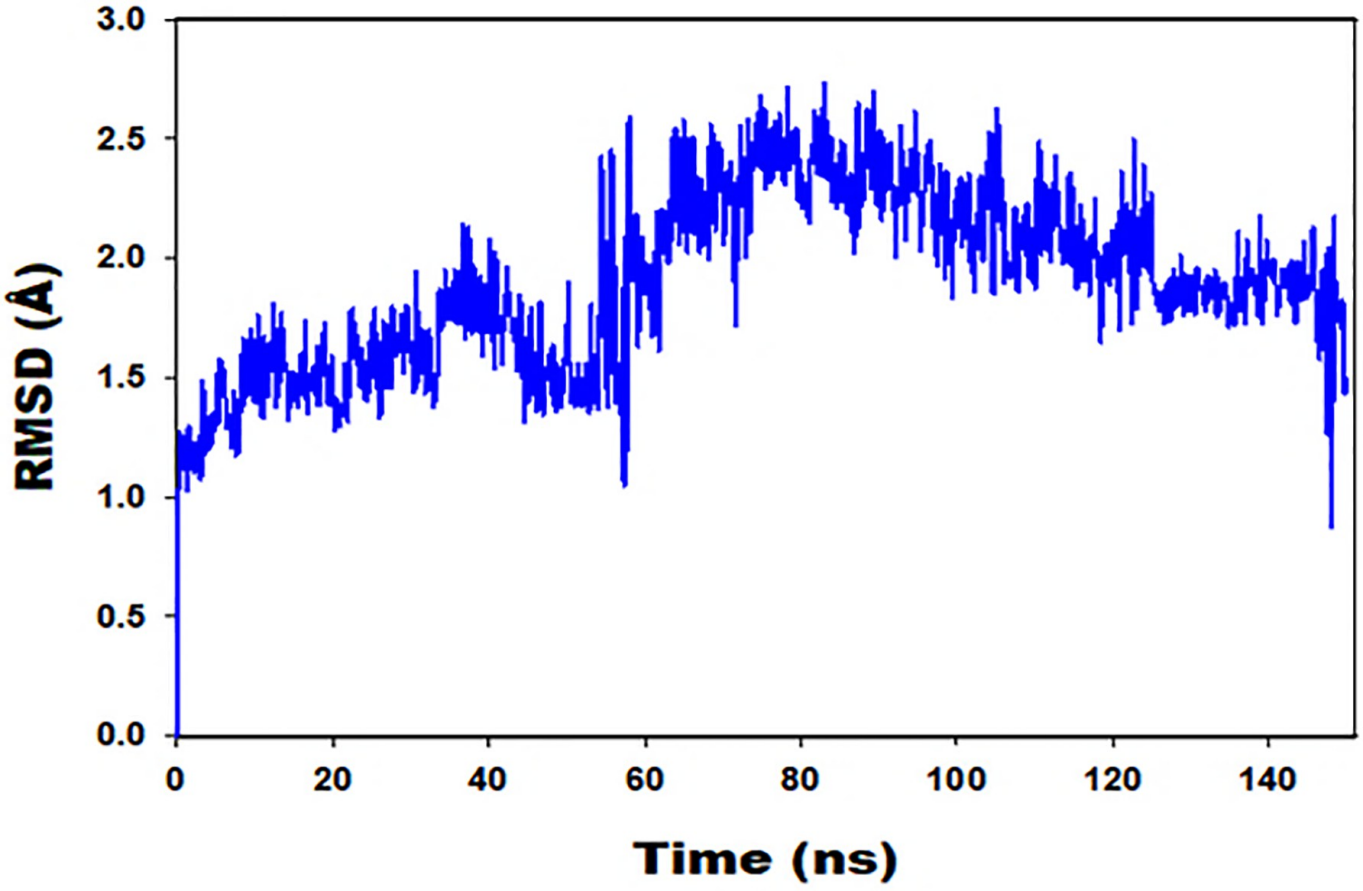
MD simulation trajectory analysis of Root Mean Square Divisions (RMSD) of Bromocriptinemesylate bound with TLR4 at 150 ns time frame.

The graph above displays the run result of a Root Mean Square Divisions MD simulation trajectory analysis (RMSD). Fig 2 shows that the TLR4-2-Bromoergocryptine Mesylate complex achieves stability at 35 ns. Following that, changes in RMSD values for target remain below 0.5 throughout the simulation, which is perfectly acceptable. After they have been equilibrated, the ligand fit to protein RMSD values range within 0.5 Angstrom. It Throughout the simulation, the ligands remained securely attached to the receptor’s binding site. As demonstrated in Fig 2, the RMSD values for ligand fit to protein do not fluctuate much across the simulation length, indicating that the ligands stay firmly attached to the receptor’s binding site.

**Fig 3:**
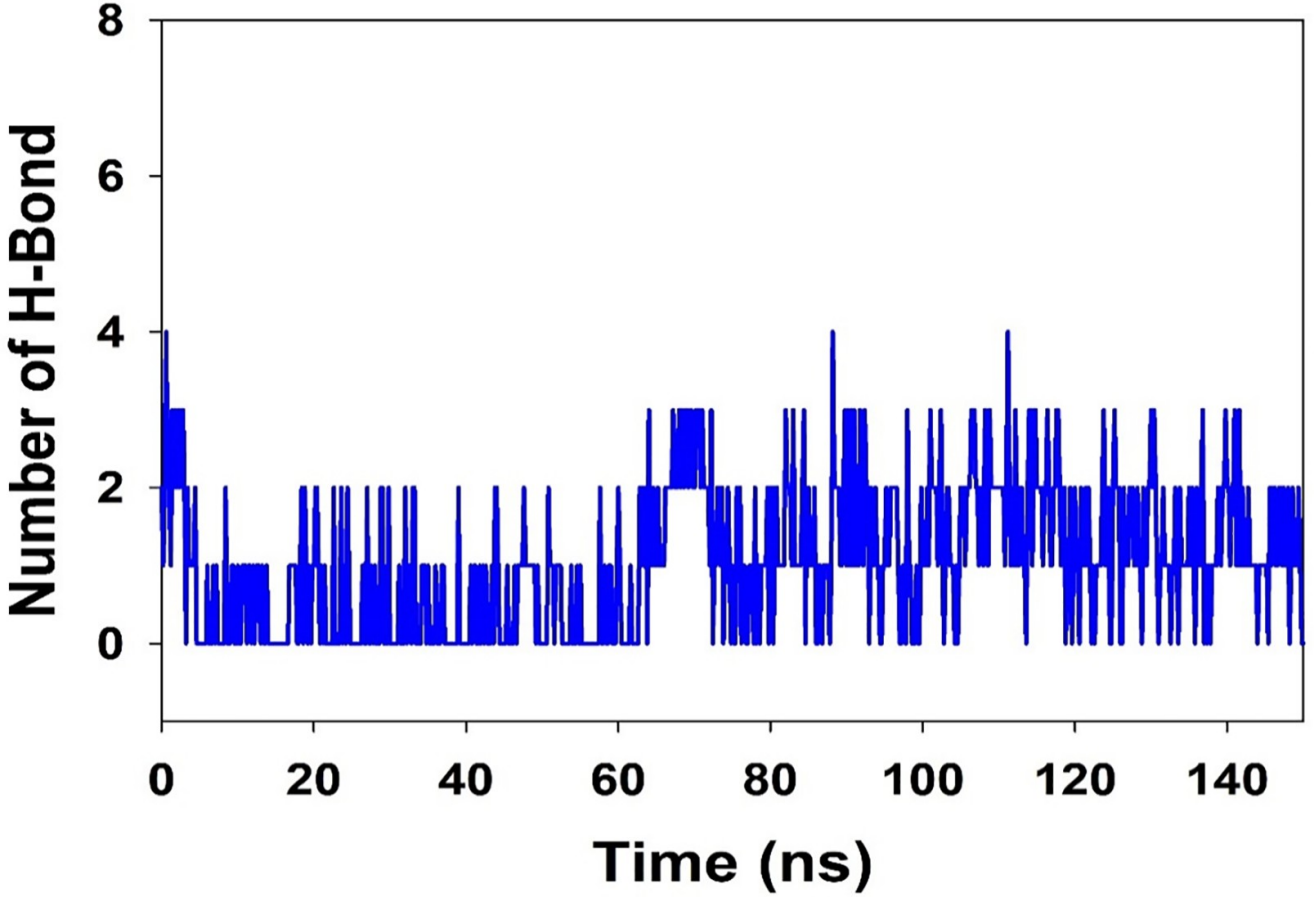
MD simulation trajectory analysis of hydrogen bonding of 2-Bromoergocryptine Mesylatebound with TLR4 at 150 ns time frame.

Fig 3 illustrates the average hydrogen bonds fashioned during the 150 ns simulation between 2-Bromoergocryptine Mesylate and the different proteins. TLR4 has two hydrogen bonds on average from 0 to 150 ns in the simulation (as illustrated in Fig 3). The 2D ligand binding graphic shows that an average of two hydrogen bonds were formed throughout the simulation. The existence of hydrogen bonds has improved the binding of TLR4 and 2-Bromoergocryptine Mesylate, making it more stable during simulation.

**Fig 4:**
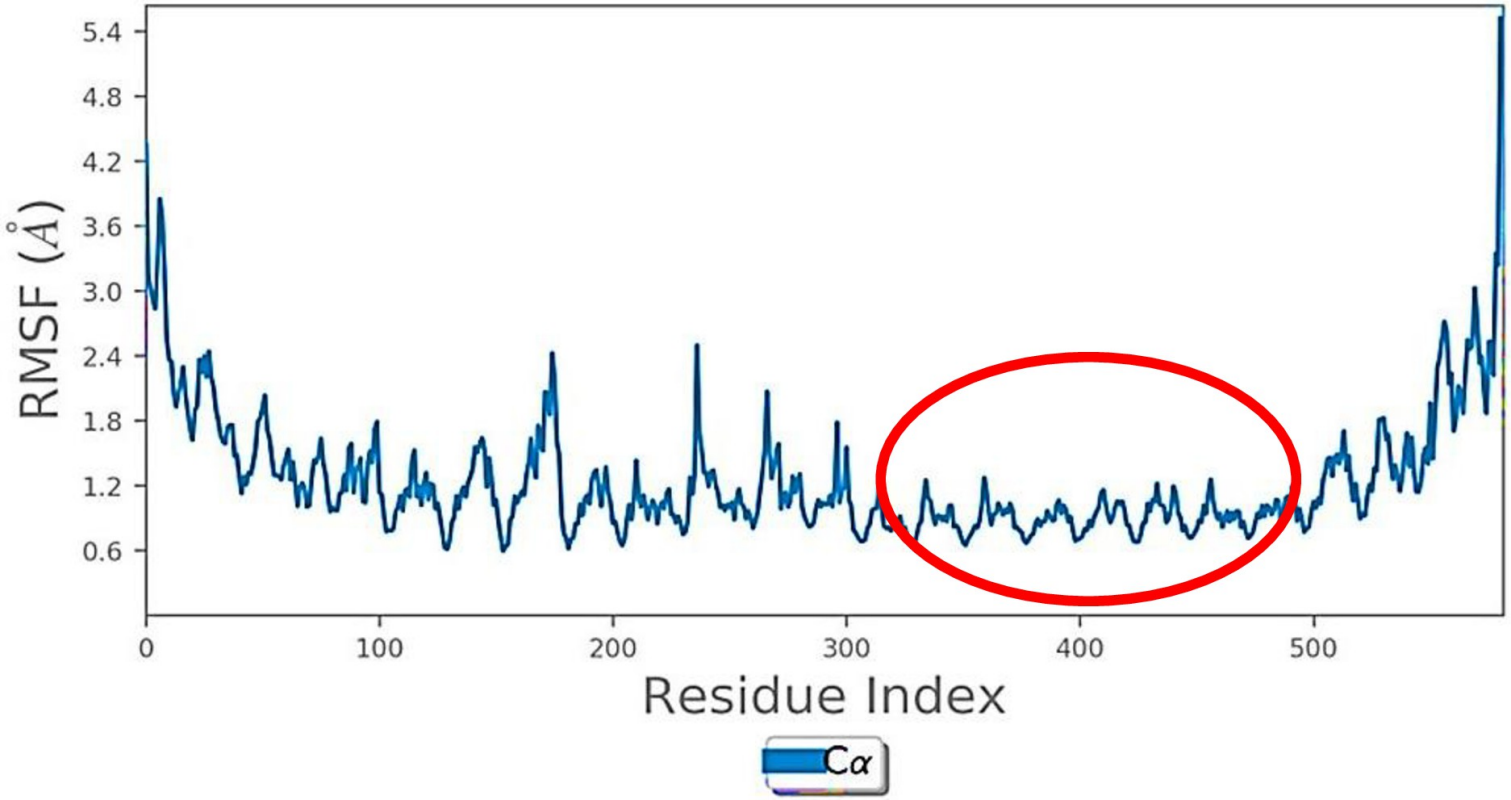
MD simulation trajectory investigation of 2-Bromoergocryptine Mesylateassociated with TLR4 RMSF at 150 ns time frame shown.

A protein’s RMSF graph shows the areas of the protein that change the most throughout the simulation. Tails (N- and C-terminal) of proteins change more often than the body of the protein, which is more stable. In comparison to the protein’s unstructured section, the alpha and beta helices and strands are more rigid and exhibit less variation. MD trajectories show that residues at the N and C-terminal regions, as well as loop areas, have the largest peaks in the trajectory (as illustrated in Fig 4).

**Fig 5:**
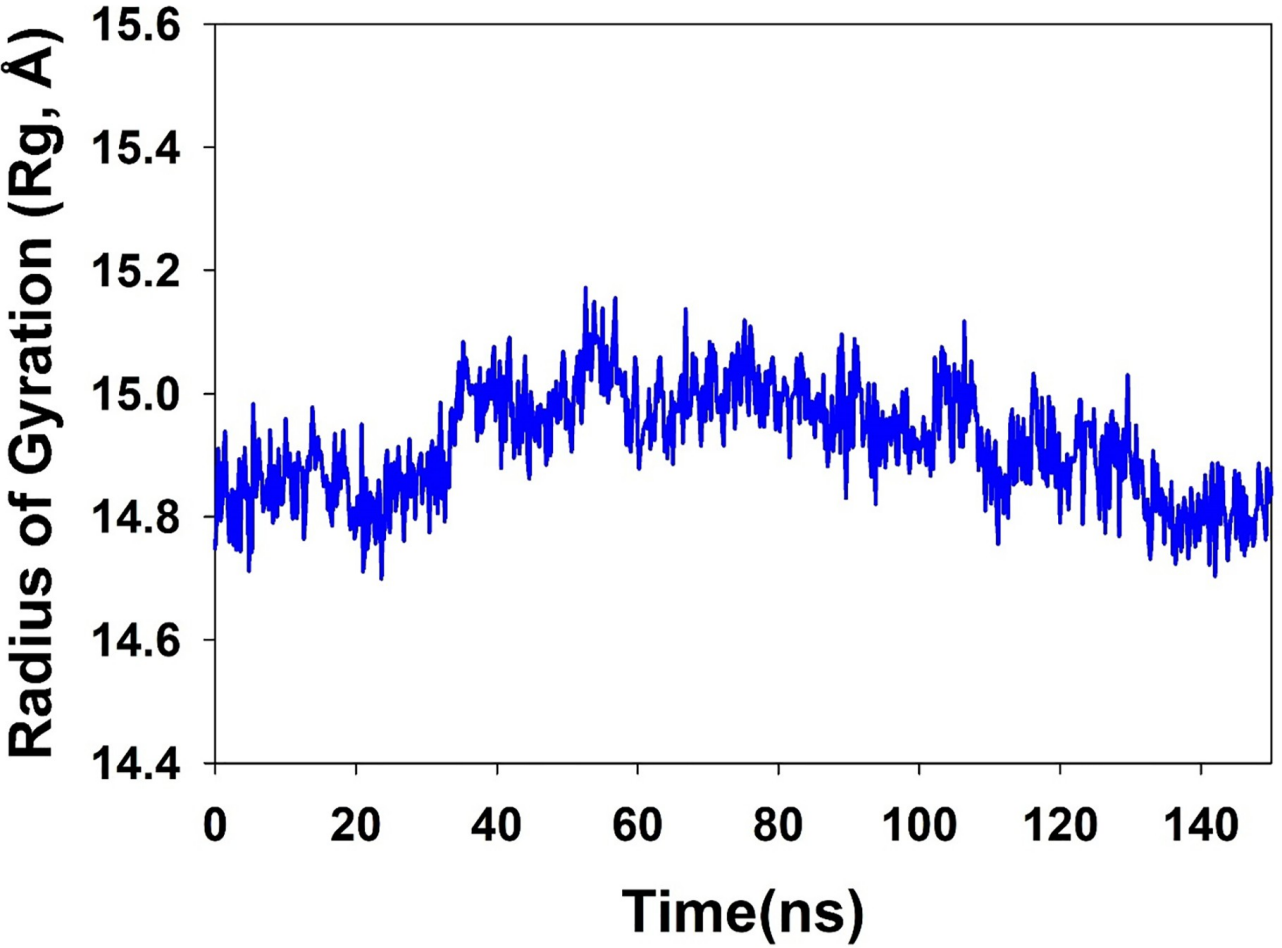
MD simulation trajectory analysis of Radius of Gyration (RoG) of 2-Bromoergocryptine Mesylatebound with TLR4 at 150 ns time frame displayed.

The compactness of the proteins may be measured by something called the radius of gyration. The Radius of Gyration of TLR4 proteins that were bound to 2-Bromoergocryptine Mesylate was shown to be decreased (illustrated in Fig 5). It can be deduced from the overall quality analysis that was performed using RMSD and Rg that the 2-Bromoergocryptine Mesylate that was bound to the protein targets posthumously in the binding cavities and that plays a significant role in maintaining the stability of the proteins possesses a fluctuation of 0.299 angstroms.

It’s conceivable that protein interactions with the ligand will become obvious throughout the simulation. The graphs that follow demonstrate how these transactions might be categorised and summarized according to the nature of the transaction. The four kinds of interactions that may occur between proteins and ligands are hydrogen bonds, hydrophobic interactions, ionic interactions, and water bridges.

**Fig 6:**
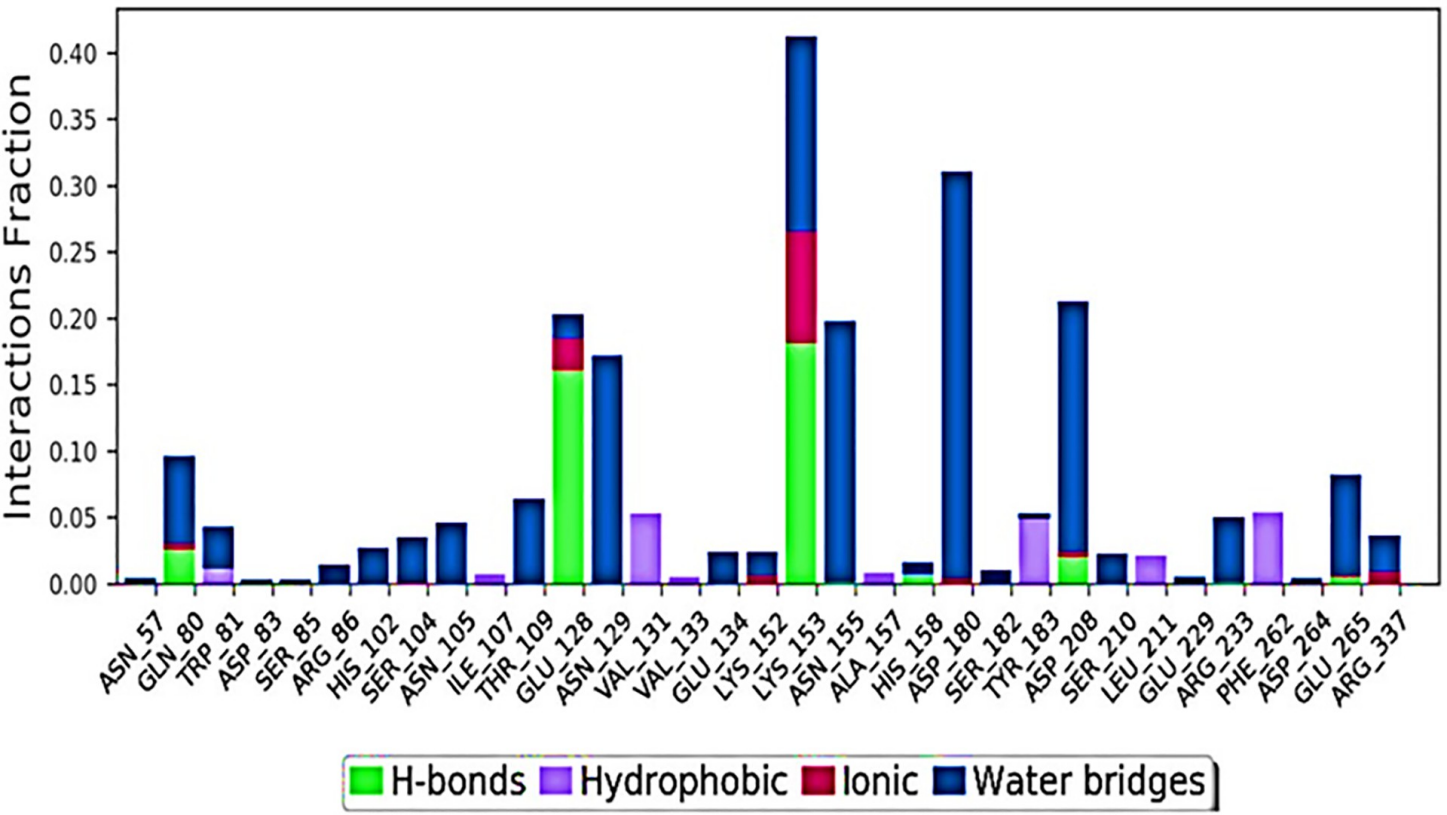
Protein-ligand contact histogram (H-bonds, hydrophobic, ionic, water bridges) of TLR4 and 2-Bromoergocryptine Mesylate.

Using Maestro’s "Simulation Interactions Diagram" panel, you may investigate the many potential permutations of each interaction type. Throughout the journey, the stacked bar charts are kept in the same configuration. Because individual protein residues may engage in several interactions with the same ligand subtype, values greater than 1.0 are not impossible to attain. The bulk of important ligand–protein interactions found by MD include hydrogen bonds and interactions involving hydrophobic forces (as depicted in Fig 6).

**Fig 7:**
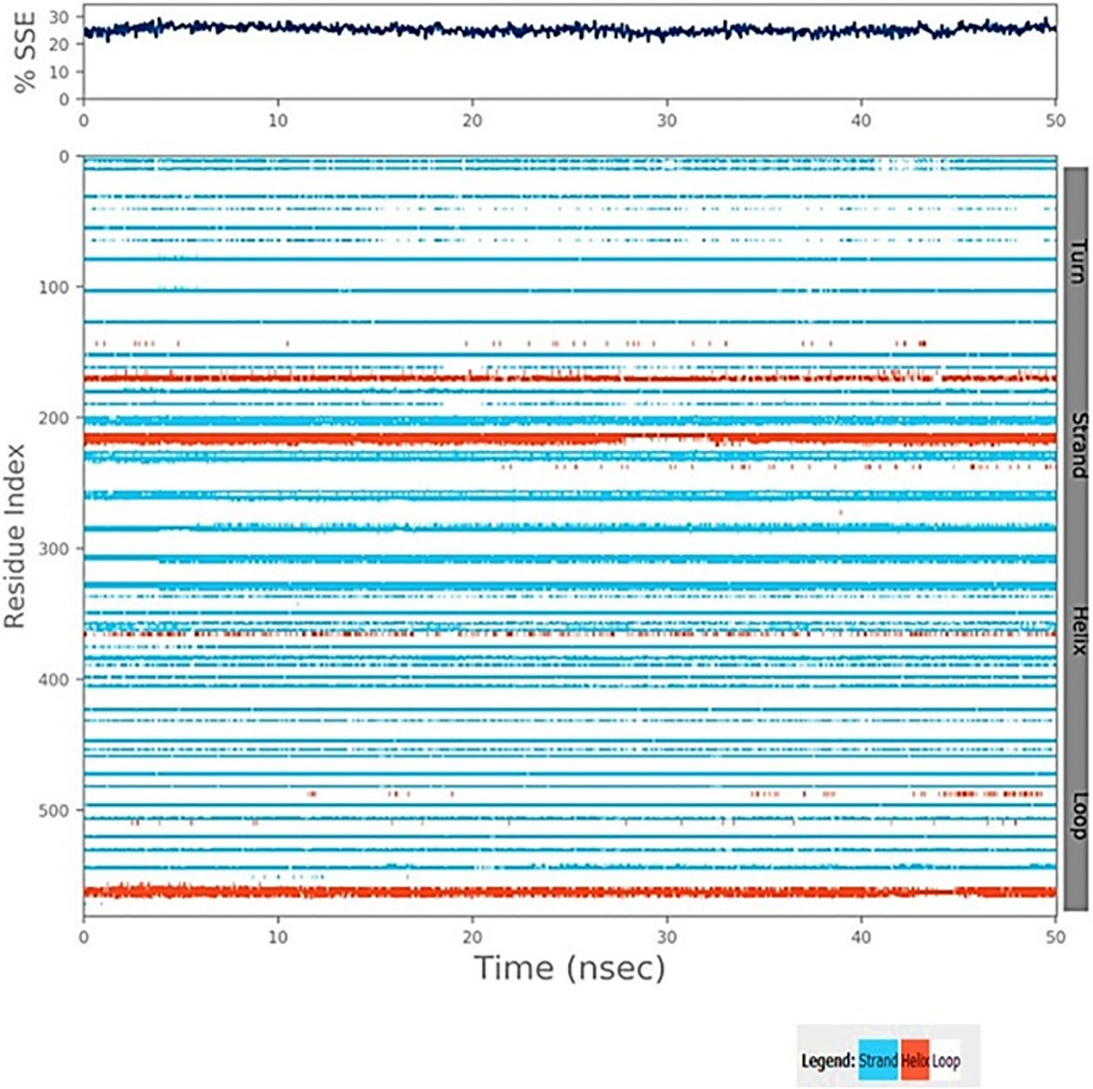
Secondary structure element distribution by residue index throughout the protein structure. Red indicates alpha helices, and blue indicate beta-strands of TLR4 and 2-Bromoergocryptine Mesylate.

Fig 7 illustrates the interactions between individual ligand atoms and protein residues. The selected trajectory (0.00 to 150.0 ns) is shown for interactions that occur more than 30% of the simulation time.

## Conclusion & discussions

Plant-based therapies are gaining popularity worldwide. Two-thirds of Western pharmaceuticals are made from natural materials. Ayurveda, the ancient Indian medical system, has meticulously recorded an astounding number of plant species [22]. Besides, our research hints to a potential for widespread commercial use of wheat ergot fungus-derived and modified2-Bromoergocryptine Mesylate.

Higher levels of inflammatory markers in the blood of people with AD indicate inflammation is present in the early stages of the disease. An accumulation of events leading to neurodegeneration begins when Aß binds to TLR4. Inflammatory processes, such as the production of chemokines and cytokines, are involved. TLR4 is linked to the onset of both diabetes and AD. Multiple diabetes-related clinical concerns, as well as the internal environment and the milieu of the brain, have been linked to TLR4 activation. Additional diabetes-related clinical problems have been linked to TLR4-mediated chronic inflammation. Current antidiabetic medicines are being reanalyzed using cutting-edge computational approaches for their potential as TLR4 inhibitors. Overall, our results imply that 2-bromoergocryptine mesylate has the potential to generate a concrete binding association in the active region of the TLR4 protein. The quality of its interaction with the protein was used to reach this conclusion. Improved glucose control in type 2 diabetics can be achieved with a healthy diet, regular exercise, and 2-bromoergocryptine mesylate. Even though being overweight increases one’s risk of developing type 2 diabetes, this is the case. In addition to these characteristics, it is also an agonist for the D2 dopamine receptor and has antidyskinetic, antiprolactinomic, and antidiabetic effects. Patients with type 2 diabetes can now choose from a wide variety of oral hypoglycemic drugs designed to normalise glucose levels in the plasma.

But they haven’t found a way to keep being successful in the long run. Using quick release has a lot of benefits, but here are just a few: The benefits of using 2-bromoergocryptine Mesylate to treat diabetes include, but are not limited to, restoring normal sleep patterns, working in a different way than the diabetes medicines that are currently used, and working well with other ways to deal with the disease. But one of these benefits is not resetting the circadian rhythm. Because it is only taken once a day in the morning instead of twice a day like other diabetic medicines, there is less chance of heart problems like heart attack and stroke. It’s easier to remember because you only have to take it once a day, in the morning. Because of these promising results, 2-Bromoergocryptine Mesylate could change the way type 2 diabetes and maybe even AD are treated.

## Supporting information

S1 Fig Energy plot of protein ligand complex system during the entire simulation event of 100 ns. The total energy (black), van der Waal’s energy (green) and coulomb energy (red) of the entire system indicating the stability of the individual systems.
(DOCX)

S1 Table Raw data file for MD simulation study. (CSV)
