# Supplementary material for: Wheat ergot fungus-derived and modified drug for inhibition of intracranial aneurysm rupture due to dysfunction of TLR-4 receptor in Alzheimer’s disease
Source: PLoS One. 2023 Jan 19;18(1):e0279616. doi: 10.1371/journal.pone.0279616 (PMC9851541; doi:10.1371/journal.pone.0279616)
Supplement: S1 Fig — The total energy (black), van der Waal’s energy (green) and coulomb energy (red) of the entire system indicating the stability of the individual systems. (DOCX) [file pone.0279616.s001.docx]

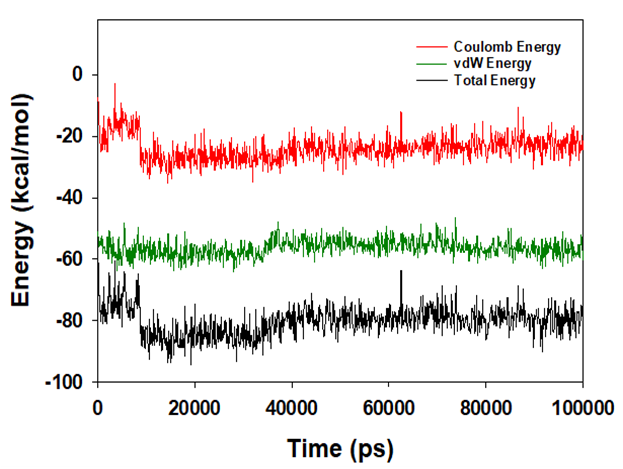


**Figure S1:** Energy plot of protein ligand complex system during the entire simulation event of 100 ns. The total energy (black), van der Waal’s energy (green) and coulomb energy (red) of the entire system indicating the stability of the individual systems.
